# No Association of a Set of Candidate Genes on Haloperidol Side Effects

**DOI:** 10.1371/journal.pone.0044853

**Published:** 2012-10-15

**Authors:** Antonio Drago, Ina Giegling, Martin Schäfer, Annette M. Hartmann, Hans-Jürgen Möller, Diana De Ronchi, Hans H. Stassen, Alessandro Serretti, Dan Rujescu

**Affiliations:** 1 Department of Psychiatry, Ludwig Maximilians University, Munich, Germany; 2 Institute of Psychiatry, University of Bologna, Bologna, Italy; 3 Psychiatric University Hospital, Zurich, Zurich, Switzerland; University of Wuerzburg, Germany

## Abstract

We previously investigated a sample of patients during an active phase of psychosis in the search for genetic predictors of haloperidol induced side effects. In the present work we extend the genetic association analysis to a wider panel of genetic variations, including 508 variations located in 96 genes. The original sample included 96 patients. An independent group of 357 patients from the CATIE study served as a replication sample. Outcomes in the investigation sample were the variation through time of: 1) the ESRS and UKU total scores 2) ESRS and UKU subscales (neurologic and psychic were included) related to tremors and 3) ESRS and UKU subscales that do not relate to tremors. Outcome in the replication sample was the presence vs absence of motoric side effects from baseline to visit 1 (∼ one month of treatment) as assessed by the AIMS scale test. Rs2242480 located in the CYP3A4 was associated with a different distribution of the UKU neurologic scores through time (permutated p = 0.047) along with a trend for a different haloperidol plasma levels (lower in CC subjects). This finding was not replicated in the CATIE sample. In conclusion, we did not find conclusive evidence for a major association between the investigated variations and haloperidol induced motoric side effects

## Introduction

Schizophrenia is a chronic devastating illness with a world wide prevalence of about one percent. Its treatment includes the use of antipsychotics, a class of drugs that antagonize, among others, the dopaminergic 2 receptors. Haloperidol is a benchmark drug for the pharmacological treatment of Schizophrenia. The genetic background of its effect and side effects remains to be clarified even though sensitivity to antipsychotics' induced side effects varies in the general population, and genetics may play a role in creating such differences [1]. In particular, knowing in advance who will develop intolerable side effects would help avoid the premature discontinuation of the pharmacological treatment which would result in a better prognosis [2]. A list of genetic variations has been associated with side effects caused by antipsychotics. A review can be found at [3]. In particular, the dopaminergic 2 receptor seems to play a major role [4]. Further, some recent genome-wide investigations widened the number of molecular pathways and candidate genes that could be of prime relevance in determining movement disorders after treatment with antipsychotics. Alkelai and colleagues [5] investigated a sample from ‘The Clinical Antipsychotic Trial of Intervention Effectiveness’ (CATIE) (n tot = 397 – in that analysis - , output was the score from the Simpson-Angus Scale (SAS) for Parkinsonism [6]. Three genes were associated with motoric side effects: EPF1, NOVA1 and FIGN. EPF1 product is a transcription factor that controls neurogenesis in the CNS [7], and it can be crucial in the migration of dopaminergic neurons to the substantia nigra [8]. FIGN product is a chaperon, a protein involved in pro-surviving events. The NOVA1 product is the neuro-oncological ventral antigen 1. Aberg and colleagues [9] analyzed a larger sample from the CATIE (n = 738 in that second analysis, output was the score from the following tests: SAS for Parkinsonism, the Barnes Akathisia Rating Scale (BARS) [10] and the abnormal involuntary movement scale (AIMS)(www.servier.com/App_Download/Neurosciences//AIMS.pdf) and brought convincing evidence that a zink protein (ZNF202) plays a role in determining the risk for motoric side effects induced by antipsychotics. This gene codes for a transcription suppressor which is particularly active in lipidic related genes. Aberg and colleagues argued that ZNF202 could play a role in the synthesis of myelin.

Tardive dyskinesia has been extensively investigated by pharmacogenetic studies as a severe and potentially irreversible motoric side effect inducted by antipsychotics. A complete and recent review can be found at [11]. Acute and potentially reversible motoric side effects induced by antipyschotics received much less attention, even though their incidence may dramatically decrease adherence to treatment, which leads to a poorer prognosis. Table 1 reports the main genetics findings published insofar. One of the most frequently investigated gene was the CYP2D6, a gene that encodes a member of the cytochrome P450 superfamily of enzymes. The cytochrome P450 proteins are monooxygenases which catalyze many reactions involved in drug metabolism and synthesis of cholesterol, steroids and other lipids. This protein localizes to the endoplasmic reticulum and is known to metabolize as many as 20% of commonly prescribed drugs, included many psychotropic drugs. The gene is highly polymorphic in the population; certain alleles result in the poor metabolizer phenotype, characterized by a decreased ability to metabolize the enzyme's substrates. On this basis, a number of pharmacogenetic investigations were conducted to find out whether variations in this gene that resulted into different drug plasma concentrations could interfere with the risk of developing an acute motoric side effect after antipsychotic treatment. Despite highly rational, this approach did not provide consistent evidence of involvement of the CYP2D6 polymorphisms in the incidence of motoric side effects incuded by antipsychotics (Table 1). The reason for that may be found in both the biological complexity and the caveats of the studies that dealt with this gene. There are many other proteins that may balance a decreased function of the CYP2D6, thus blunting the pharmacogenetic effect of this variation. For example the human multidrug resistance gene (MDR1) codes for a P-glycoprotein that acts as a an ATP-dependent efflux transporter that protects the central nervous system from environmental toxins and xenobioticsmay. Variations located in the MDR1 may modulate the functionality of the coded protein that may balance the hypo/hyper activity of the CYPD26. Thus, studies that did not take into account the variations located in the CYP2D6 and the MDR1 may suffer from a relevant biological stratification factor. Consistently with this, two variations located in the gene that code for the MDR1 (Table 1) were found to be associated with dystonia and akathisia in a sample of 56 Caucasian patients treated with risperidone [12]. Further, the medium-small samples enrolled for the analyses of the CYPD2D6 variations (Table 1) decreased the power of the studies, so that minor genetic effects passed undetected. The serotonin trasporter (5-HTT) [13], and the dopamine transporter (DAT1) [14] were also investigated as potential modulators of antipsychotic induced side effects. Interestingly, Guzey and colleages reported that the DAT1 VNTR polymorphism and the dopamine receptor D2 (DRD2) Taq1A A1 associated with the DAT1 VNTR 9 repeat alleles were associated with higher risk of extrapyramidal side effects in a sample of 119 Caucasian patients treated with different antipsychotics. This finding is particularly interesting in that the DAT1 regulates the dopamine turnover and the DRD2 is one of the main molecular targets of antipychotics, the blockage thereof is considered to be central to the development of motoric side effects [11]. Consistently, the Ser9Gly variant of the dopaminergic receptor D3 gene (DRD3) was also found to be involved as a risk factor in the incidence of motoric side effects after antipsychotic treatment [15]. Nevertheless, the role of mutations located in dopaminergic receptors was not confirmed in a large sample of 665 patients [16]. Different study designs and medications could be at the basis of this lack of replication. We could not find a genetic association between the short/long promoter variation in the serotonin trasnporter and antipsychotic induced motoric side effects, but the study was exploratory with a small sample (47 subjects) to hold enough power to detect minor genetic effects [13]. The serotoninergic system was nevertheless proved to hold a potential for being a mediator of motoric effects induced by antipsyhotics. The serotonin receptors 2C and 2A were investigated in the same study we conducted for analysing the impact of the serotonin transporter variations. Despite the sample was small, we could identify an impact of the 102C allele of HTR2A and the −697C and 23Ser alleles of HTR2C which were more frequent among patients with extrapyramidal side effects [13]. This finding could not be replicated in a larger sample though [14], and more research is needed to clarify the impact of the serotoninergic system towards antipsychotic induced motor side effects. Antipsychotic induced parkinsonism was associated with a multiallelic variation in ATP1A3 gene in a study that involved 156 Caucasian patients [17]. ATP1A3 codes for a P-type cation transport, an ATPase integral membrane protein responsible for establishing and maintaining the electrochemical gradients of Na and K ions across the plasma membrane.

**Table 1:** Previous genetic association findings in literature for acute motoric symptoms induced by antipsychotics.

Gene/variation	author	sample	study design	antipsychotic	p	finding
5HTTLPR	18573584	56 Caucasian patients	Case-control	Haloperidol/risperidone	ns	no association
ATP1A3; UniSTS:155910	21072501	156 Caucasian patients	Case-control	various	0.019	parkinsonism was associated with a multiallelic variation in ATP1A3 gene
CYP2D6	20599499	50 Caucasian patients	Case-control	risperidone	0.006	CYP2D6 genotype was also associated with the average plasma concentration of risperidone active moiety which was associated with dystonia and parkinsonism
CYP2D6	11214775	119 American patients	Case-control	various	ns	no association
CYP2D6	9068770	76 Caucasian patients	Case-control	various	ns	no association
CYP2D6	12960748	320 Asiatic patients	Case-control	haloperidol	ns	no association
DRD2 −241A>G −141cins TaqIB Taq ID Val90Ala Pro141Ser Set311cys Taq1A (310) Cys(311) Ala(96)	12192613	665 Caucasian patients	Case-control	various	ns	no association
DRD2 Taq1A, Ser311Cys, −141CIns/Del; DRD3 Ser9Gly; HTR2A 102C, His452Tyr; HTR2C −697C and 23Ser	17102980	47 Caucasian	Case-control	various	0.01<p<0.02	The 102C allele of HTR2A and the −697C and 23Ser alleles of HTR2C were more frequent among patients with EPS
DRD2 Taq1A; DRD3 Msc1; DAT1 VNTR; 5-HT(2A) 102T/C, His452Tyr, 516 C/T, and Thr25Asn; 5HTTLPR	17225991	119 Caucasian	Case-control	various	0.030<p<0.040	DRD2 Taq1A polymorphism was significantly higher in the EPS group along with the DAT1 VNTR polymorphism and the DRD2 Taq1A A1 associated with the DAT1 VNTR 9 repeat alleles
DRD3 Ser9Gly	10893495	150 Caucasian	Case-control	various	0.002	Homozygosity for the Ser9Gly variant of the DRD3 gene was connected to a higher incidence of akathysia
MDR1 C3435T and G2677T/A	20060871	59 Caucasian patients	Case-control	risperidone	0.013<p<0.042	G2677T/A and C3435T associations with dystonia and akathisia

Overall, evidence gathered in so far is not consistent enough to provide the basis for a genetic tool to predict who will develop side effects when taking antipsychotic drug treatments [1]. Further research is then required. In the present paper we focus on motoric side effects caused by haloperidol in a sample of acutely ill psychotic in patients previously investigated both from a clinical [18], [19] and a genetic [18] point of view. We also replicated our findings on a public available genome-wide database from the NIMH funded CATIE (Clinical Antipsychotic Trials in Intervention Effectiveness) [20].

## Materials and Methods

We used a sample for investigation (n tot = 101, n with complete genetic data = 96, psychotic patients treated with haloperidol) and a sample for replication (n tot = 357, psychotic patients from the CATIE study).

### Sample for investigation

#### Characteristics of the sample for investigation

This sample has been described in detail elsewhere [19]. Briefly, 96 (42 females) acutely ill patients of Caucasian descendant were treated with haloperidol and assessed for a period of one month. Patients were enrolled at the Department of Psychiatry, Ludwig-Maximilian-University of Munich, Germany. Exclusion criteria were not having signed the informed consent for the study, a known contraindication for treatment with haloperidol, tardive dyskinesia, severe neurological or medical disorders, organic brain diseases, pregnancy and acute suicidality. Furthermore, patients were excluded if they received comedications, such as beta blockers, antidepressants, or benzodiazepines with a possible influence on the antipsychotic treatment and its side effect. Patients were treated with haloperidol during the acute phase of the illness and then shifted to another antipsychotic treatment in case of lack of response or severe side effects. Diagnosis was obtained through the SCID interview and psychopathological measurements administered by two psychiatrists with reliable inter-rater evaluation results (k>0.80). Haloperidol plasma levels, the Udvalg for Kliniske Undersøgelser side effect rating scale (UKU) [21] and Extrapyramidal Symptom Rating Scale (ESRS) [22] scores were assessed more than weekly during the first month of treatment. The study was approved by the ethics committee of the Ludwig-Maximilian University and carried out in accordance with the ethical standards laid down in the 1964 Declaration of Helsinki and later revisions. A format for the written consent is attached to the present application. For being included in the study, patients had do give written consent to their participation. All potential participants who declined to participate or otherwise did not participate were eligible for treatment (if applicable) and were not disadvantaged in any other way by not participating in the study.

### Characteristics of the replication sample

The CATIE (Clinical Antipsychotic Trials for Intervention Effectiveness) Schizophrenia Trial was a National Institute of Mental Health (NIMH) funded large, double-blind trial that compared the effectiveness of five antipsychotics in the treatment of schizophrenia. These drugs included four atypical SGAs (second generation antipsychotcis: olanzapine, quetiapine, risperidone, and ziprasidone, all introduced since 1994) and perphenazine (a medium-potency, conventional FGA (first generation antipsychotics) available since the 1950s). Fourteen hundred sixty adults with schizophrenia were studied for up to 18 months. Diagnoses were determined by Structured Clinical Interview for DSM-IV (Diagnostic and Statistical Manual of Mental Disorders, fourth edition). The study was a pragmatic hybrid of efficacy and effectiveness trial designs [23]. Ages ranged from 18 to 65. Inclusion criteria allowed patients with comorbid medical, psychiatric, and substance abuse problems to participate in the study, consistent with “real world” design. Patients were also excluded if they had a diagnosis of schizoaffective disorder, mental retardation, or other cognitive disorders, as were those who were treatment resistant (defined as persisting severe symptoms despite adequate trials of one of the study medications or prior treatment with clozapine). Patients with severe or unstable medical conditions and pregnant women were excluded. Study participants provided written informed consent following discussion of the study and formal testing to demonstrate understanding of its elements [20]. The CATIE trial consisted of three phases. Data from the phase 1 served for replication in the present article.

In Phase 1, participants were randomly assigned to double-blind treatment of up to 18 months either with one of the four SGAs or with perphenazine. Patients with TD (tardive dyskinesia) were not administered perphenazine. The primary outcome measure of the study was all-cause treatment discontinuation, but a number of tests were administered to patients, including the AIMS which served for replication in our sample. Phase 1 examined 1460 intent-to-treat subjects. Genome-wide data was available for 410schizophrenia patients as retrieved - upon request – from https://www.nimhgenetics.org/available_data/schizophrenia/pgc/pgc_sz1.php. . Inclusion criteria from the original CATIE study were having been treated with a first generation antipsychotic before entering the study and being not missing in the outcome measures. The first inclusion criteria was set in order to match the cases in the group for replication with the cases in the original group. Subjects were then analyzed irrespectively of the kind of antipsychotic they were administered. The rational for this approach is that motoric side effects from a first generation antipsychotic may appear weeks after its introduction. Thus, we included all subjects treated with a first generation antipsychotic at baseline and followed them for ∼ a month. On the other hand, the introduction of a second generation antipsychotic may decrease the antipsychotic induced motoric side effects. Then the clinical baseline scores were employed only when scores at visit 1 were missing.

### Outcome in the sample for investigation

Primary outcome in the sample for investigation was the variation over time of the sum of ESRS [22] items related to tremors (ESRS questionnaire item 7 = ‘Tremors, shaking’; ESRS parkinsonism item 5: ‘tremor right arm’,’tremor left arm’, ‘tremor right leg’,‘tremor left leg’,‘tremor head’,‘tremor jaws’,‘tremor tongue’,‘tremor lips’), and the UKU [21] neurologic and autonomic scores. Tremors are included in the UKU neurologic subscales and were analyzed separately. Missing values were handled by a last observation carried forward analysis.

### Clinical covariates in the sample for investigation

Haloperidol plasma levels were the clinical covariate in the sample under investigation, based on the results of a previous analysis of the same sample [24]. Haloperidol plasma levels were not included in the model as covariates when the genes pharmacokinetically related to haloperidol were analyzed.

### Outcome in the replication sample

Outcomes in the replication sample were dichotomic and corresponded to a ≥1 score at the SEVSCORE (AIMS [22] sums of scores from 1 to 7), BRSOBJ (Barnes akathisia scale [10], sum of scores from 1 to 3) and EPSMEAN (Simpson-Angus Scale Movement Score [6], mean of items 1 to 6) at visit 1. For EPSMEAN a threshold for defining presence/absence of side effects was the median of the distribution of the variable. When any of these scores were missing, a score ≥1 at the Movement Severity Score at baseline was used instead. All the variables were available and no further edited from the original data.

### Predictors

A list of 508 variations were analyzed as predictors of the outcomes in the investigation sample. 508 variations located in known candidate genes were selected for the analysis. Candidates included genes involved in cell-to-cell and cell-to-matrix interaction (ADAM22 for example), glutamatergic and GABAergic related genes (GRIA4 and GABRA4 for example) and genes related to the pharmacokinetics of antipsychotics (e.g. CYP2D6).

The most significant findings were investigated as predictors in the CATIE sample in order to reduce the probability of false positive findings. The CATIE sample was imputed to allow the replication of the same variants in both samples. These genes extended a panel of about one hundred variations that were previously investigated in the same sample [18]. Supplementary material reports the total of investigated variations in the present analysis (Table S1).

### Genotyping in the sample for investigation

#### Genotyping

SNPs were chosen in order to balance the maximum gene coverage and the costs of the analysis. A medium of 5 variations for each gene were included in the analysis.

DNA extraction was done with the QIAamp Blood Maxi Kit (QIAamp DNA Blood Midi/Maxi Handbook, Firma Qiagen, Hilden, Germany, 2005). DNA concentration was adjusted using the PicoGreen quantitation reagent (Invitrogen, Karlsruhe, Germany). SNPs were selected from the NCBI SNP database http://www.ncbi.nlm.nih.gov/entrez/query.fcgi?db=snp), pubmed publications (http://www.ncbi.nlm.nih.gov/entrez/query.fcgi?db=PubMed) and “Tagger” (http://www.broad.mit.edu/mpg/tagger/server.html). Only validated SNPs with a minor allele frequency of more than 1% were selected. All SNPs were in Hardy–Weinberg Equilibrium (p<0.01). 12,5 ng DNA were genotyped using the iPLEX assay on the MassARRAY MALDI-TOF mass spectrometer (SEQUENOM, Hamburg, Germany). Genotyping call rates in cases and controls were all >96%. Allele frequencies were similar to CEU sample frequencies (www.hapmap.org).

The list of genes and SNPs investigated is reported in Supplementary materials.

### Genotyping in the replication sample

We employed the public available NIMH CATIE dataset. Individual genotypes were downloaded from the net. As for the the methods in the original study, individual genotyping was conducted by Perlegen Sciences (Mountain View, CA, USA) using three genotyping chips: Affymetrix 500 K ‘A’ chipset (Nsp I and Sty I chips; Santa Clara, CA, USA) and a custom 164 K chip created by Perlegen [25] to provide additional genome coverage, further details can be found at [26].

### Data imputation in the replication sample

Imputation procedure was conducted in PLINK (Purcell et al. 2007), source for imputation was the hapmap_CEU_r23a dataset (http://pngu.mgh.harvard.edu/~purcell/plink/res.shtml.). To speed up the procedures, only the parts of the genome harboring the variations found to be associated with outcome in the investigation sample were imputed. A quality threshold for imputation of 0.95 was required.

### Statistics

Repeated measures ANOVA was the test of choice to study the association between genotypes and primary outcome in the sample of primary investigation. The analysis of clinical covariates was conducted in previous publications [27]. Haloperidol plasma levels were not included as covariates when genes related to the pharmacokinetics of haloperidol were analyzed as predictors, in that the haloperidol plasma levels would be an effect rather than a confounder of such genes' activity. Time was included as a continuous variable and the gene*time effect was investigated. A logistic regression between the outcome (presence/absence of motoric side effects) and the predictors (best SNPs from the primary analysis) was the test of choice in the replication sample. All non genome-wide analyses were run in R [28], dedicated packages. For the analyses that implicated the investigation of part of the genome-wide data we employed Plink [29]. We had sufficient power (0.80) to detect a medium effect size (∼0.5) between three groups (three genotypes) each composed by 33 subjects on average.

## Results

Sample characteristics of the investigation sample are reported in table 2. Table 3 reports the distribution of the outcome in the primary sample. Table 4 reports the significant different distribution of the outcome in the primary sample according to the rs2242480 genetic variability. Table 5 reports the characteristics of the sample for replication.

**Table 2:** Sample clinical characteristics.

Variable	Result	Association with the outcome (p value)
Sex	M = 50; F = 46TOT = 96	0.65
Age (yrs) (mean±SD)	34.23±11.55	0.12
Age at onset (yrs) (mean±SD)	28.43±9.58	0.07
Ethnicity	Caucasian = 96	/

**Table 3:** ESRS and UKU scores distribution through time in the discovery sample.

	ESRS				
	ESRS total	ESRS parkinsonism	ESRS dystonia	ESRS dyskinesia	ESRS questionnaire
Day 1	0.06±0.64	0.01±0.1	0.01±0.18	0.62±1.41	0.02±0.21
Day 03	15.20±17.75	5.11±7.28	3.56±6.37	2.30±3.71	4.22±4.69
Day 07	14.37±18.54	7.60±10.29	1.37±4	2.20±3.24	3.77±4.37
Day 14	15.10±17.03	8.90±11.36	0.54±2.2	2.37±2.51	3.81±3.80
Day 21	15.24±16.47	8.58±9.67	0.03±0.26	2.23±2	3.21±2.96
Day 28	14.40±15.90	7.76±8.29	/	/	3.33±2.89

	UKU		
	UKU total	UKU neurological	UKU psychic
Day 1	7.78±4.46	0.20±0.89	7.26±4.31
Day 03	8.50±5.79	2.89±3.23	5.60±4.29
Day 07	7.44±5.84	2.97±3.38	4.41±3.92
Day 14	7.20±5.59	3.26±3.26	3.93±3.72
Day 21	6.47±4.58	2.97±2.53	3.54±3.26
Day 28	5.9±4.01	2.86±2.51	3.03±2.49
	Haloperidol plasma levels (microg/L)		
Day 1	3.51±4.42		
Day 03	6.65±7.24		
Day 07	7.69±9.82		
Day 14	6.61±4.54		
Day 21	7.10±5.74		
Day 28	7.15±6.11		

**Table 4:** UKU neurological scores haloperidol oral doses and plasma levels for CYP3A4 rs2242480.

	UKU neurological scores		Haloperidol oral doses		Haloperidol plasma levels	
rs2242480	CT	CC	CT	CC	CT	CC
Day 1	0.125±0.5	0±0	/	/	1.95±1.34	4.09±4.97
Day 03	3.56±2.44	2.41±2.94	/	/	7.22±6.48	6.91±7.77
Day 07	4.37±3.70	2.17±2.56	12.46±6.44	10.46±5.06	8.88±5.53	7.71±11.12
Day 14	4.68±3.45	2.41±2.40	10.97±6.74	10.35±5.98	9.47±6.47	6.13±3.82
Day 21	4.81±3.37	2.28±2.12	10.23±8.09	10.68±6.05	11.88±7.73	6.57±4.83
Day 28	4.75±3.35	2.20±2.06	10.38±7.86	10.25±7.25	12.50±11.75	6.28±3.70

/ = not detected.

**Table 5:** characteristics of the replication sample.

Variable	Result	Association with the outcome (p value)
Age	41.66±11.39	0.25
Gender	Females = 77 Males = 280 tot = 357	0.17
Ethnicity	White = 357	/
motoric side effects at visit 1 or at baseline if visit 1 was missing	AIMS: yes = 138 (38%) Barnes: yes = 142 (39%) EPS (median; mean±sd) = 0.16; 0.40±1.14	/

Rs2242480 located in CYP3A4 influenced the distribution of the UKU neurological scores through time in the sample for primary analysis (table 4). Moreover, it had an appreciable though not significant effect on haloperidol plasma levels. There were 77 homozygotes for the C allele at rs2242480, 21 heterozygotes and 1 homozygote for the T allele. 8 subjects were missing. Analyses were conducted again including the CC and CT genotypes only. The final association with the distribution of UKU neurological scores resulted robust (p = 5e-4; F = 7.68; df = 2) to survive 100000 permutations (permutated p = 0.047) (table 4) but not a Bonferroni correction (p = 9.8e-5). No other association survived the permutation, either from the principal or the secondary outcome. This finding was not replicated in the CATIE sample. The lack of replication mandate cautiousness in interpreting this result. No haplotype analyses was then conducted based on the finding involving the rs2242480 to avoid multitesting and possible false positive findings. Supplementary material contains the characteristics and gentotypic distribution of the variations under analysis.

## Discussion and Conclusions

CYP3A4 is involved in the pharmacokinetics of haloperidol [30] and its activity is induced by this drug [31]. Of note, CYP3A4 is expressed also by the the blood brain barrier (BBB) and by neurons [32]. A prosurviving activity of this enzyme in neuron was consistently recently demonstrated [32].

Rs2242480 located in CYP3A4 was found to interfere with the UKU neurological scores in a way which was not completely accounted by the haloperidol plasma levels in the sample.

Intriguingly then, rs2242480 harbored by CYP3A4 could dampen the ability of the BBB to stop haloperidol before it enters the neuronal milieu or also the ability of neurons to handle the toxic products of haloperidol metabolism [33]. Carriers of the CC genotype would have either a lower neuro/blood haloperidol level ratio, or their neurons would be more active in metabolizing the neurotoxic products of haloperidol. The first hypothesis could be objected because there was no effect of this variation in the distribution of PANSS scores in the sample. Nevertheless, this finding did not survive a Bonferroni correction as was not replicated in the CATIE sample. Thus, the probability of a false positive finding despite the biological rationale of this finding is very high.

A long list of genes and variations were included in this analysis. The present panel of investigation contained both genes that have been investigated in candidate associations (dopamine 2 receptor for example) and genes that belong to molecular pathways whose activity is consistent with the results from the genome-wide investigations. For example, we included a list of variations harbored by ADAM22 whose product is involved in cell-cell and cell-matrix interactions, and some transcription factors such as PRDM2. The rational for such inclusion is consistent with the proliferation and migration of neurons to the substantia nigra, which has been suggested to be a pivotal molecular event for antipsychotic induced movement disorders [5].

Nevertheless, no other significant association beside the CYP3A4 was found in this analysis. This could be dependent on the small sample size which prevented the identification of small genetic effects towards the investigated phenotypes, or on the incomplete gene coverage. On the other hand, the analysis of relevant variables such as the haloperidol plasma levels are more feasible in smaller samples. This may be relevant in the search of a better balance between the required sample size and the required detail of clinical variables to detect the effects of the genetic variations. Finally, some relevant genetic variants such as the copy number variations or the epigenetic control were not investigated.

In conclusion, we found a variation harbored by the CYP3A4 gene with a trend of significant association with the UKU neurologic scores distributionEven though rs2242480 was associated with a different distribution of haloperidol plasma levels in the sample (table 4), this finding was not statistically significant. Thus, the effects of this variation may be related also to a different ability of neurons to handle higher levels of haloperidol. Then, it is possible to hypothesize that the rs2242480 located in CYP3A4 may impact on the resilience of neurons to haloperidol toxicity but further research is needed. Rs2242480 is located in the intron region of NM_001202855.2 but it is very closed to an exonic region (∼20 b (http://www.ncbi.nlm.nih.gov/projects/SNP/snp_ref.cgi?rs=2242480)). Thus, it could play a role in impacting the expression rate of the CYP3A4 by influencing the molecular process at the boundary between the exonic and intronic region. Consistently, this variation proved to be significantly associated with adverse reactions to metadone [34] and with coronary disease [35]. The lack of replication in the CATIE sample may be attributable to the imputation analysis which could have potentially affected the replication results and also to the clinical characteristics of the sample which are not fully consistent with the investigation sample. Moreover, the CATIE design aimed at addressing compliance to the treatment more than addressing the treatment efficacy, so that the comparison between the sample for investigation and the sample for replication we used may have suffered from relevant formal caveats.

## Supporting Information

Table S1 **Table S1 reports the complete list of investigated variations.**
(DOC)
