# Supplementary material for: No Association of a Set of Candidate Genes on Haloperidol Side Effects
Source: PLoS One. 2012 Oct 15;7(10):e44853. doi: 10.1371/journal.pone.0044853 (PMC3471928; doi:10.1371/journal.pone.0044853)
Supplement: Table S1 — reports the complete list of investigated variations. (DOC) [file pone.0044853.s001.doc]

Supplementary material

Table S1. Genes and variations under investigation

| gene_names | rs_names | minor allele frequency |
| --- | --- | --- |
| PRDM2 | rs10803425 | T=0.286 |
| PRDM2 | rs17393663 | T=0.229 |
| PRDM2 | rs1203678 | T=0.332 |
| PRDM2 | rs2014788 | A=0.49 |
| PRDM2 | rs2245197 | A=0.207 |
| PRDM2 | rs2697971 | A=0.197 |
| OPRD1 | rs4654322 | C=0.278 |
| OPRD1 | rs204055 | T=0.407 |
| OPRD1 | rs680090 | C=0.49 |
| OPRD1 | rs508448 | C=0.455 |
| GRIK3 | rs530817 | G=0.111 |
| GRIK3 | rs4652931 | T=0.302 |
| GRIK3 | rs1160751 | G=0.405 |
| GRIK3 | rs3753776 | T=0.379 |
| GRIK3 | rs2134846 | T=0.392 |
| CYP4B1 | rs632233 | G=0.237 |
| CYP4B1 | rs2405335 | C=0.162 |
| CYP4B1 | rs4646485 | G=0.348 |
| CYP4B1 | rs3766200 | G=0.292 |
| CYP4B1 | rs2297809 | A=0.144 |
| CYP2J2 | rs10789082 | C=0.24 |
| CYP2J2 | rs1155002 | T=0.414 |
| CYP2J2 | rs7515289 | G=0.245 |
| CYP2J2 | rs11572204 | G=0.237 |
| CYP2J2 | rs3754206 | A=0.219 |
| ADAR | rs3766927 | A=0.209 |
| ADAR | rs2229857 | A=0.209 |
| APOB | rs693 | T=0.449 |
| APOB | rs10199768 | T=0.414 |
| APOB | rs520354 | G=0.479 |
| APOB | rs589566 | T=0.489 |
| POMC | rs6713532 | C=0.253 |
| POMC | rs7565877 | G=0.083 |
| POMC | rs934778 | C=0.255 |
| POMC | rs1009388 | G=0.219 |
| CYP1B1 | rs2855658 | A=0.402 |
| CYP1B1 | rs2551188 | T=0.294 |
| CYP1B1 | rs2567206 | A=0.294 |
| GPR39 | rs7592134 | G=0.278 |
| GPR39 | rs2241764 | A=0.273 |
| GPR39 | rs13402545 | A=0.46 |
| GPR39 | rs3109134 | C=0.362 |
| GPR39 | rs4547577 | C=0.444 |
| LYPD1 | rs1131671 | A=0.394 |
| LYPD1 | rs17396537 | C=0.412 |
| LYPD1 | rs17396607 | C=0.343 |
| LYPD1 | rs2435612 | C=0.343 |
| LYPD1 | rs1430630 | A=0.337 |
| LYPD1 | rs2566537 | C=0.408 |
| LYPD1 | rs2248193 | T=0.338 |
| GAD1 | rs11542313 | C=0.392 |
| GAD1 | rs769390 | C=0.182 |
| GAD1 | rs701492 | T=0.216 |
| GAD1 | rs7578661 | G=0.214 |
| STAT1 | rs3771300 | A=0.474 |
| STAT1 | rs1547550 | C=0.338 |
| STAT1 | rs2280234 | T=0.359 |
| STAT1 | rs7562024 | T=0.363 |
| STAT1 | rs10208033 | C=0.448 |
| STAT4 | rs925847 | T=0.273 |
| STAT4 | rs16833215 | G=0.305 |
| STAT4 | rs12463658 | C=0.404 |
| STAT4 | rs1551443 | T=0.342 |
| CYP27A1 | rs4674338 | A=0.418 |
| CYP27A1 | rs7594289 | G=0.423 |
| CYP27A1 | rs4674341 | G=0.47 |
| CYP27A1 | rs4674344 | T=0.479 |
| CYP27A1 | rs6709815 | G=0.49 |
| SLC4A3 | rs12993807 | A=0.036 |
| SLC4A3 | rs2289782 | C=0.237 |
| SLC4A3 | rs2305055 | G=0.247 |
| SLC4A3 | rs635311 | A=0.258 |
| SLC4A3 | rs684428 | A=0.201 |
| SLC4A3 | rs2305054 | T=0.207 |
| SLC4A3 | rs619540 | C=0.212 |
| SLC6A1 | rs1710873 | C=0.423 |
| SLC6A1 | rs2697150 | T=0.247 |
| SLC6A1 | rs1710892 | T=0.384 |
| SLC6A1 | rs2933308 | A=0.376 |
| SLC6A1 | rs2933307 | G=0.448 |
| SLC6A1 | rs1062246 | G=0.394 |
| SLC6A6 | rs4685155 | A=0.352 |
| SLC6A6 | rs748098 | G=0.47 |
| SLC6A6 | rs6766142 | G=0.495 |
| SLC6A6 | rs7611075 | A=0.409 |
| SLC6A6 | rs3773162 | A=0.343 |
| CHMP2B | rs904115 | A=0.111 |
| CHMP2B | rs2279720 | A=0.056 |
| CHMP2B | rs3828364 | A=0.418 |
| CHMP2B | rs13061754 | T=0.469 |
| DRD3 | rs2399496 | T=0.485 |
| DRD3 | rs2134655 | A=0.278 |
| DRD3 | rs9825563 | G=0.348 |
| GABRA2 | rs573400 | G=0.388 |
| GABRA2 | rs483160 | A=0.387 |
| GABRA2 | rs279842 | C=0.378 |
| GABRA2 | rs1442060 | A=0.489 |
| GABRA2 | rs2119767 | A=0.245 |
| GABRA4 | rs6843462 | A=0.428 |
| GABRA4 | rs16859794 | C=0.444 |
| GABRA4 | rs2229940 | A=0.348 |
| GABRB1 | rs10010460 | T=0.421 |
| GABRB1 | rs6824550 | G=0.455 |
| GABRB1 | rs1442098 | T=0.121 |
| GABRB1 | rs4695201 | C=0.402 |
| GABRB1 | rs1076015 | A=0.474 |
| GABRB1 | rs10440176 | G=0.424 |
| GABRB1 | rs6289 | G=0.25 |
| CLOCK | rs1047354 | G=0.359 |
| CLOCK | rs10462028 | A=0.328 |
| CLOCK | rs6834676 | C=0.38 |
| CLOCK | rs11937487 | G=0.357 |
| CLOCK | rs3805146 | T=0.362 |
| CLOCK | rs12501746 | T=0.291 |
| CLOCK | rs11942279 | C=0.356 |
| CLOCK | rs11133379 | G=0.361 |
| CLOCK | rs1522106 | C=0.369 |
| CLOCK | rs12510990 | C=0.358 |
| CLOCK | rs11945370 | G=0.351 |
| CLOCK | rs3736544 | A=0.369 |
| CLOCK | rs6824955 | T=0.368 |
| CLOCK | rs4864994 | C=0.354 |
| CLOCK | rs11240 | G=0.263 |
| CLOCK | rs12651640 | A=0.359 |
| CLOCK | rs4580704 | G=0.372 |
| CLOCK | rs13128582 | C=0.374 |
| CLOCK | rs6817267 | G=0.353 |
| CLOCK | rs11931061 | G=0.372 |
| CLOCK | rs2140077 | A=0.369 |
| CLOCK | rs12506788 | T=0.361 |
| CLOCK | rs11133388 | C=0.356 |
| CLOCK | rs17722870 | G=0.258 |
| CLOCK | rs12642716 | A=0.354 |
| CLOCK | rs17722979 | A=0.268 |
| CLOCK | rs9997288 | T=0.365 |
| CLOCK | rs11735267 | C=0.362 |
| CLOCK | rs6858803 | C=0.356 |
| CLOCK | rs7684810 | C=0.367 |
| CLOCK | rs939823 | T=0.357 |
| CLOCK | rs2070062 | C=0.268 |
| CLOCK | rs7677085 | T=0.357 |
| CLOCK | rs17085842 | C=0.359 |
| CLOCK | rs12510681 | T=0.356 |
| CLOCK | rs12500162 | C=0.361 |
| CLOCK | rs3805154 | C=0.369 |
| CLOCK | rs3805155 | C=0.361 |
| CLOCK | rs2177129 | C=0.368 |
| CLOCK | rs7686261 | G=0.365 |
| CLOCK | rs6837710 | C=0.369 |
| CLOCK | rs3817444 | A=0.368 |
| CLOCK | rs10462033 | T=0.273 |
| CLOCK | rs13108499 | C=0.361 |
| CLOCK | rs10016506 | T=0.369 |
| CLOCK | rs13116194 | A=0.357 |
| CLOCK | rs13117836 | T=0.357 |
| CLOCK | rs6554285 | C=0.268 |
| CLOCK | rs6554286 | G=0.358 |
| CLOCK | rs11133397 | A=0.354 |
| CLOCK | rs10005989 | T=0.391 |
| CLOCK | rs4864548 | A=0.357 |
| CLOCK | rs11133399 | G=0.366 |
| CLOCK | rs4865010 | C=0.361 |
| GRID2 | rs992995 | G=0.306 |
| GRID2 | rs7663835 | A=0.416 |
| GRID2 | rs7672245 | A=0.402 |
| GRID2 | rs5019028 | G=0.265 |
| GRID2 | rs6819321 | T=0.449 |
| PGRMC2 | rs3755896 | A=0.343 |
| MTNR1A | rs6847693 | C=0.18 |
| MTNR1A | rs13140444 | C=0.409 |
| MTNR1A | rs13113549 | G=0.414 |
| SLC6A3 | rs40184 | A=0.464 |
| SLC6A3 | rs11133767 | A=0.323 |
| SLC6A3 | rs27048 | T=0.429 |
| SLC6A3 | rs250686 | G=0.454 |
| SLC6A3 | rs2455391 | T=0.207 |
| HTR1A | rs10085024 | G=0.474 |
| HTR1A | rs1423691 | C=0.479 |
| HTR1A | rs878567 | C=0.469 |
| HTR1A | rs6295 | C=0.469 |
| GRIA1 | rs548294 | A=0.419 |
| GRIA1 | rs716517 | C=0.258 |
| GRIA1 | rs707176 | C=0.316 |
| GRIA1 | rs11953799 | T=0.293 |
| GRIA1 | rs1461224 | C=0.465 |
| ADRA1B | rs3729604 | A=0.141 |
| ADRA1B | rs1474190 | G=0.193 |
| ADRA1B | rs6892282 | T=0.369 |
| ADRA1B | rs6888306 | T=0.207 |
| ADRA1B | rs13171967 | T=0.195 |
| GABRA1 | rs7734447 | G=0.474 |
| GABRA1 | rs6894357 | A=0.469 |
| GABRA1 | rs7701394 | T=0.374 |
| GABRA1 | rs4259206 | A=0.475 |
| GABRA1 | rs12716387 | T=0.374 |
| GABRA1 | rs2279020 | G=0.385 |
| GABRG2 | rs183294 | T=0.333 |
| GABRG2 | rs365054 | T=0.437 |
| TNF | rs909253 | C=0.224 |
| TNF | rs1799964 | C=0.192 |
| TNF | rs1800629 | A=0.126 |
| TNF | rs361525 | A=0.025 |
| TNF | rs3093661 | A=0.02 |
| TNF | rs1800610 | T=0.162 |
| TNF | rs3093662 | G=0.051 |
| TNF | rs3093664 | G=0.051 |
| TNF | rs3093665 | C=0.025 |
| NEU1 | rs574914 | T=0.153 |
| NEU1 | rs9267649 | A=0.126 |
| NEU1 | rs13118 | A=0.061 |
| NEU1 | rs4947332 | T=0.01 |
| NEU1 | rs521977 | T=0.211 |
| NEU1 | rs660594 | G=0.364 |
| NEU1 | rs644827 | T=0.371 |
| NOTCH4 | rs3134939 | A=0.419 |
| NOTCH4 | rs3131290 | A=0.48 |
| NOTCH4 | rs422951 | A=0.475 |
| NOTCH4 | rs443198 | C=0.387 |
| NOTCH4 | rs3096702 | T=0.313 |
| HLADRB1 | rs2308765 | A=0.247 |
| GRM4 | rs2499679 | G=0.222 |
| GRM4 | rs10947476 | T=0.374 |
| GRM4 | rs2499712 | G=0.439 |
| GRM4 | rs744102 | C=0.367 |
| GRM4 | rs2499730 | G=0.389 |
| HTR1B | rs6296 | C=0.306 |
| HTR1B | rs6298 | T=0.308 |
| HTR1B | rs130058 | T=0.283 |
| HTR1B | rs11568817 | G=0.424 |
| HTR1B | rs17273700 | G=0.423 |
| GABRR1 | rs368873 | G=0.247 |
| GABRR1 | rs10944436 | A=0.48 |
| GABRR1 | rs2183797 | G=0.399 |
| GABRR1 | rs12192394 | T=0.286 |
| GABRR1 | rs914479 | G=0.308 |
| GABRR2 | rs282129 | T=0.247 |
| GABRR2 | rs4707535 | T=0.173 |
| GABRR2 | rs745013 | T=0.09905 |
| GABRR2 | rs2325202 | G=0.367 |
| GABRR2 | rs7744352 | A=0.469 |
| MCHR2 | rs12215494 | T=0.402 |
| MCHR2 | rs13195863 | A=0.153 |
| MCHR2 | rs4840106 | A=0.286 |
| MCHR2 | rs9376634 | G=0.4 |
| MCHR2 | rs7754794 | T=0.367 |
| GRIK2 | rs9498663 | A=0.273 |
| GRIK2 | rs4839801 | T=0.434 |
| GRIK2 | rs13202221 | G=0.363 |
| GRIK2 | rs2852620 | T=0.395 |
| GRM1 | rs2073287 | G=0.495 |
| GRM1 | rs1331639 | A=0.49 |
| GRM1 | rs9386147 | T=0.293 |
| GRM1 | rs56566 | C=0.247 |
| GRM1 | rs6923492 | T=0.484 |
| TBP | rs12717 | G=0.419 |
| TBP | rs6456230 | T=0.48 |
| TBP | rs7765023 | G=0.48 |
| TBP | rs1042327 | T=0.439 |
| TBP | rs2072916 | A=0.443 |
| CRHR2 | rs4722999 | C=0.312 |
| CRHR2 | rs3779250 | G=0.354 |
| CRHR2 | rs255105 | T=0.327 |
| CRHR2 | rs255119 | G=0.333 |
| ABCB1 | rs1045642 | C=0.49 |
| ABCB1 | rs4148737 | G=0.414 |
| ABCB1 | rs1202172 | G=0.316 |
| ABCB1 | rs13233308 | T=0.48 |
| ABCB1 | rs10246878 | A=0.204 |
| ABCB1 | rs2157929 | C=0.068 |
| ADAM22 | rs2279542 | G=0.495 |
| ADAM22 | rs1637499 | A=0.475 |
| ADAM22 | rs2022163 | C=0.47 |
| ADAM22 | rs10282572 | C=0.485 |
| ADAM22 | rs7793308 | C=0.379 |
| ADAM22 | rs9641023 | G=0.474 |
| CYP3A5 | rs4646457 | C=0.073 |
| CYP3A5 | rs15524 | C=0.067 |
| CYP3A5 | rs6976017 | A=0.035 |
| CYP3A5 | rs28365094 | C=0.111 |
| CYP3A5 | rs4646453 | T=0.035 |
| CYP3A5 | rs4646450 | T=0.167 |
| CYP3A5 | rs776746 | A=0.071 |
| CYP3A5 | rs28365067 | A=0.053 |
| CYP3A5 | rs28371764 | T=0.025 |
| CYP3A7 | rs2687087 | G=0.095 |
| CYP3A7 | rs2687081 | G=0.098 |
| CYP3A7 | rs10211 | G=0.095 |
| CYP3A7 | rs12360 | C=0.097 |
| CYP3A7 | rs999900026 | A=0.061 |
| CYP3A4 | rs2037498 | G=0.092 |
| CYP3A4 | rs2037499 | C=0.094 |
| CYP3A7 | rs2687142 | T=0.092 |
| CYP3A4 | rs2687136 | G=0.098 |
| CYP3A4 | rs2404955 | A=0.111 |
| CYP3A4 | rs3735451 | G=0.117 |
| CYP3A4 | rs6956344 | T=0.117 |
| CYP3A4 | rs2242480 | T=0.103 |
| CYP3A4 | rs4646437 | T=0.141 |
| CYP3A4 | rs2246709 | G=0.281 |
| CYP3A4 | rs2740574 | G=0.02 |
| LRRN3 | rs7800862 | G=0.167 |
| LRRN3 | rs2301733 | A=0.399 |
| LRRN3 | rs17158333 | C=0.194 |
| ADRA1A | rs1048101 | C=0.428 |
| ADRA1A | rs7821620 | C=0.273 |
| ADRA1A | rs13261054 | T=0.313 |
| ADRA1A | rs556793 | G=0.352 |
| ADRA1A | rs476631 | C=0.263 |
| ADRA1A | rs1383914 | A=0.495 |
| NRG1 | rs999900017 | T=0.408 |
| NRG1 | rs10954809 | T=0.495 |
| NRG1 | rs1487145 | G=0.379 |
| NRG1 | rs2976527 | T=0.242 |
| CRH | rs11997416 | A=0.068 |
| CRH | rs6996265 | A=0.078 |
| CRH | rs6982394 | A=0.035 |
| CRH | rs28364018 | G=0.061 |
| CRH | rs12721510 | T=0.071 |
| CRH | rs3176921 | C=0.081 |
| CRH | rs6999780 | A=0.081 |
| CRH | rs1870393 | C=0.172 |
| DBH | rs1611114 | T=0.321 |
| DBH | rs1611115 | T=0.156 |
| DBH | rs2873804 | T=0.49 |
| DBH | rs1541333 | G=0.49 |
| DBH | rs77905 | T=0.495 |
| DBH | rs732833 | G=0.374 |
| DBH | rs129882 | T=0.227 |
| CYP2C18 | rs1575934 | G=0.434 |
| CYP2C18 | rs12243416 | A=0.212 |
| CYP2C18 | rs7914753 | G=0.434 |
| CYP2C18 | rs2860840 | T=0.359 |
| CYP2C19 | rs12248560 | T=0.217 |
| CYP2C19 | rs7916649 | A=0.434 |
| CYP2C19 | rs4917623 | T=0.469 |
| CYP2C19 | rs4617515 | A=0.432 |
| CYP2C9 | rs4918758 | C=0.343 |
| CYP2C9 | rs4918766 | A=0.348 |
| TH | rs2070762 | T=0.5 |
| TH | rs4074905 | G=0.291 |
| TH | rs4930043 | T=0.258 |
| TH | rs6356 | A=0.414 |
| TH | rs7925375 | T=0.217 |
| TH | rs11042962 | C=0.459 |
| TPH1 | rs10741734 | G=0.449 |
| TPH1 | rs2056246 | T=0.427 |
| TPH1 | rs172423 | C=0.253 |
| TPH1 | rs10488682 | T=0.258 |
| SLC6A5 | rs7942430 | C=0.295 |
| SLC6A5 | rs1945771 | A=0.328 |
| SLC6A5 | rs11025673 | G=0.418 |
| SLC6A5 | rs2276433 | T=0.352 |
| BDNF | rs6265 | A=0.232 |
| BDNF | rs11030101 | T=0.479 |
| BDNF | rs10835210 | A=0.432 |
| BDNF | rs7104207 | T=0.469 |
| BDNF | rs7127507 | C=0.242 |
| MTNR1B | rs8192552 | A=0.082 |
| MTNR1B | rs10765576 | A=0.384 |
| MTNR1B | rs12290860 | A=0.036 |
| MTNR1B | rs1562444 | G=0.432 |
| GRIA4 | rs11226805 | T=0.47 |
| GRIA4 | rs1445617 | T=0.449 |
| GRIA4 | rs10128540 | T=0.407 |
| GRIA4 | rs661329 | C=0.432 |
| GRIA4 | rs1144410 | T=0.49 |
| GRIA4 | rs3758790 | C=0.392 |
| NCAM | rs2850302 | A=0.369 |
| NCAM | rs686050 | C=0.378 |
| NCAM | rs584427 | A=0.434 |
| NCAM | rs2303377 | C=0.369 |
| NCAM | rs605843 | G=0.235 |
| NCAM | rs618114 | A=0.389 |
| DRD2 | rs1800497 | T=0.184 |
| DRD2 | rs6279 | C=0.372 |
| DRD2 | rs6277 | T=0.449 |
| DRD2 | rs1116313 | C=0.49 |
| DRD2 | rs12364051 | G=0.495 |
| DRD2 | rs7131056 | A=0.344 |
| HTR3B | rs4938056 | T=0.447 |
| HTR3B | rs1176756 | A=0.306 |
| HTR3B | rs1176744 | G=0.307 |
| HTR3B | rs3782025 | C=0.479 |
| HTR3A | rs1062613 | T=0.232 |
| HTR3A | rs1985242 | A=0.337 |
| HTR3A | rs11214796 | C=0.199 |
| HTR3A | rs10160548 | G=0.321 |
| HTR3A | rs1150220 | T=0.194 |
| HTR3A | rs1176713 | C=0.193 |
| GRIK4 | rs6589829 | C=0.434 |
| GRIK4 | rs3133846 | C=0.268 |
| GRIK4 | rs1954787 | T=0.439 |
| GRIK4 | rs7104543 | G=0.423 |
| GRIK4 | rs2298725 | T=0.293 |
| GRIK4 | rs2298723 | C=0.4 |
| GRIN2B | rs1806191 | A=0.485 |
| GRIN2B | rs2300258 | G=0.177 |
| GRIN2B | rs7301328 | C=0.449 |
| HTR2A | rs7322347 | A=0.444 |
| HTR2A | rs9567737 | C=0.468 |
| HTR2A | rs2770304 | C=0.359 |
| GABRB3 | rs2017247 | A=0.227 |
| GABRB3 | rs11636988 | G=0.468 |
| GABRB3 | rs17646626 | A=0.438 |
| GABRB3 | rs4906902 | G=0.143 |
| GABRA5 | rs9744196 | A=0.393 |
| GABRA5 | rs7170180 | C=0.356 |
| GABRA5 | rs6606872 | C=0.49 |
| GABRA5 | rs140685 | T=0.495 |
| GABRA5 | rs17647448 | G=0.47 |
| CHRNA7 | rs883473 | T=0.296 |
| CHRNA7 | rs2175886 | C=0.439 |
| CHRNA7 | rs7175581 | A=0.387 |
| CHRNA7 | rs12438848 | T=0.426 |
| CHRNA7 | rs7170028 | T=0.49 |
| CHRNA7 | rs2337980 | T=0.463 |
| SRP14 | rs8208 | C=0.304 |
| SRP14 | rs33932994 | G=0.149 |
| SRP14 | rs8041057 | C=0.303 |
| SRP14 | rs17551891 | T=0.3 |
| SRP14 | rs7497289 | A=0.274 |
| CYP1A1 | rs4646903 | C=0.077 |
| CYP1A1 | rs1048943 | G=0.026 |
| CYP1A1 | rs1799814 | A=0.071 |
| CYP1A1 | rs4646421 | T=0.066 |
| CYP1A1 | rs2606345 | C=0.294 |
| CYP1A1 | rs2470893 | A=0.27 |
| CYP1A2 | rs762551 | C=0.258 |
| CYP1A2 | rs2472304 | G=0.318 |
| CYP1A2 | rs2470890 | C=0.316 |
| CYP1A2 | rs17861162 | G=0.045 |
| CYP1A2 | rs11854147 | T=0.288 |
| CYP1A2 | rs11072507 | G=0.316 |
| AKAP13 | rs17553006 | G=0.429 |
| AKAP13 | rs716400 | T=0.235 |
| AKAP13 | rs4843075 | G=0.294 |
| AKAP13 | rs11631018 | A=0.354 |
| SLC6A2 | rs2242446 | C=0.342 |
| SLC6A2 | rs36027 | G=0.389 |
| SLC6A2 | rs36021 | A=0.444 |
| SLC6A2 | rs4564560 | T=0.47 |
| SLC6A2 | rs2279805 | T=0.47 |
| SLC6A2 | rs2242447 | C=0.313 |
| C16orf3 | rs3743826 | A=0.364 |
| C16orf3 | rs7191175 | A=0.495 |
| C16orf3 | rs3785184 | A=0.077 |
| SLC6A4 | rs2054847 | T=0.394 |
| SLC6A4 | rs4583306 | G=0.387 |
| SLC6A4 | rs2020942 | A=0.404 |
| SLC6A4 | rs2020939 | T=0.397 |
| SLC6A4 | rs2066713 | T=0.403 |
| CACNA1A | rs4926249 | A=0.359 |
| CACNA1A | rs4499352 | G=0.454 |
| CACNA1A | rs2248069 | G=0.301 |
| CACNA1A | rs16003 | G=0.5 |
| CYP2B6 | rs8101756 | C=0.263 |
| CYP2B6 | rs10500282 | C=0.278 |
| CYP2B6 | rs8113200 | A=0.349 |
| CYP2B6 | rs11671243 | A=0.344 |
| APOE | rs440446 | C=0.359 |
| APOE | rs769449 | A=0.101 |
| APOE | rs429358 | C=0.126 |
| NLRP7 | rs269933 | T=0.455 |
| NLRP7 | rs269951 | A=0.455 |
| NLRP7 | rs4806626 | C=0.247 |
| NLRP7 | rs775882 | T=0.258 |
| NLRP7 | rs775883 | T=0.359 |
| CACNA1A | rs2419549 | A=0.362 |
| ADRA1D | rs2236554 | A=0.374 |
| ADRA1D | rs6116268 | C=0.465 |
| ADRA1D | rs1556832 | C=0.469 |
| ADRA1D | rs6133098 | C=0.283 |
| GRIK1 | rs363500 | A=0.298 |
| GRIK1 | rs1016700 | T=0.374 |
| GRIK1 | rs2178865 | G=0.362 |
| GRIK1 | rs145472 | T=0.318 |
| COMT | rs5844402 | G=0.449 |
| COMT | rs740603 | G=0.5 |
| COMT | rs4680 | G=0.449 |
| NF2 | rs715490 | C=0.268 |
| NF2 | rs2530661 | C=0.463 |
| NF2 | rs2857639 | G=0.383 |
| NF2 | rs2857650 | C=0.318 |
| NF2 | rs1034880 | G=0.318 |
| MCHR1 | rs133067 | C=0.195 |
| MCHR1 | rs133070 | G=0.354 |
| MCHR1 | rs133074 | T=0.404 |
| MCHR1 | rs9611386 | G=0.056 |
| CYP2D6 | rs5758589 | G=0.495 |
| CYP2D6 | rs28360521 | T=0.134 |
| MAOA | rs4570308 | G=0.253 |
| MAOA | rs1465108 | A=0.247 |
| MAOA | rs909525 | G=0.288 |
| MAOA | rs2283724 | G=0.286 |
| MAOA | rs6323 | G=0.273 |
| MAOA | rs1137070 | T=0.286 |
| MAOB | rs1799836 | G=0.469 |
| MAOB | rs10521432 | A=0.284 |
| MAOB | rs5905512 | G=0.5 |
| HTR2C | rs498207 | C=0.314 |
| HTR2C | rs543229 | G=0.162 |
| HTR2C | rs12688102 | G=0.323 |
| HTR2C | rs6318 | C=0.162 |
| HTR2C | rs6644090 | T=0.147 |
| GRIA3 | rs4825840 | A=0.242 |
| GRIA3 | rs2157271 | G=0.359 |
| GRIA3 | rs502434 | A=0.361 |
| GRIA3 | rs5910006 | C=0.344 |
